# First Birth after Sperm Selection through Discontinuous Gradient Centrifugation and Artificial Insemination from a Chromosomal Translocation Carrier 

**DOI:** 10.1155/2014/906145

**Published:** 2014-01-23

**Authors:** Alexandre Rouen, Capucine Hyon, Richard Balet, Nicole Joyé, Nino Guy Cassuto, Jean-Pierre Siffroi

**Affiliations:** ^1^Medical Genetics and Embryology Department, AP-HP, Trousseau Hospital, 28 avenue du Docteur Arnold Netter, 75012 Paris, France; ^2^Reproductive Medicine Department, Bluets Hospital, 4 rue Lasson, 75012 Paris, France; ^3^Drouot Laboratory, 21 rue Drouot, 75009 Paris, France

## Abstract

*Introduction*. Balanced chromosomal carriers, though usually healthy, are confronted with recurrent spontaneous abortions and malformations in the offspring. Those are related to the transmission of an abnormal, chromosomally unbalanced genotype. We evidenced that the proportion of unbalanced spermatozoa can be significantly decreased through a sperm preparation process called discontinuous gradient centrifugation (DGC). We therefore started offering intrauterine inseminations with this procedure to couples with a male translocation carriers. *Case Presentation*. We report the case of a 37-year-old man carrying a t(3;10)(q25;p13) reciprocal translocation. He and his partner had had trouble conceiving for ten years and had four spontaneous abortions. DGC in this patient decreased the proportion of unbalanced spermatozoa from 63.6% to 52.3%. They were therefore offered intrauterine insemination with DGC, which eventually led to the birth of a healthy female child carrying the paternal translocation. *Conclusion*. We showed that translocation carriers could be offered intrauterine inseminations with DGC. Before this, the only two options were natural conception with prenatal diagnosis and termination of chromosomally unbalanced fetuses or preimplantation genetic diagnosis, which is a much heavier and costly procedure. We are currently offering this option through a multicentric program in France, and this is the first birth originating from it.

## 1. Introduction

The prevalence of chromosomal translocation carriers is about 1/500 in the general population. Although these translocations are usually balanced and associated to a normal phenotype, they can lead to recurrent abortions and malformations in the offspring through the fertilization of a genetically unbalanced gamete [1]. Indeed, chromosomal translocation carriers present with a certain proportion of unbalanced gamete, spermatozoa, or oocytes, which varies between patients and which can be evaluated in male patients through fluorescent in situ hybridization (FISH) performed on spermatozoa [2]. However, there is presently no way of selecting chromosomally balanced spermatozoa prior to fertilization. No association has been shown between chromosomal content and cell morphology [3]. However, unbalanced spermatozoa have been shown to have a higher apoptosis rate than their balanced counterpart in Robertsonian and reciprocal translocations as well as in pericentric inversions [4]. This led our team to evaluate the effect of discontinuous gradient centrifugation (DGC) on the proportion of unbalanced spermatozoa. We found the overall decrease to be 38.7% for Robertsonian translocation and 22.6% for reciprocal translocations and we therefore suggested that these couples could be offered artificial insemination with DGC, even when not presenting with infertility [5].

## 2. Case Presentation

We report the case of a 37-year-old man carrying a t(3;10)(q25;p13) reciprocal translocation. The patient was recruited into the research project described above through a chromosomal disorders patient organization (Association Valentin, Eragny sur Oise, France). He and his wife, a healthy 36-year-old woman, had four miscarriages most likely related to an unbalanced transmission of the paternal translocation, although no genetic evaluation was conducted at the time. He was not considered to be infertile, since the couple had not had trouble obtaining pregnancies. This was confirmed by normal semen parameters on several occasions. The proportion of unbalanced spermatozoa was 63.6% before DGC and 52.3% after DGC. The couple was therefore offered intrauterine insemination, preceded by a DGC-based sperm preparation.

Pregnancy was obtained after the second insemination. Considering the large size of the translocated chromosomal segments, we estimated that an unbalanced karyotype in the fetus would lead to rather severe malformations, detectable by ultrasound examination. These were normal throughout the pregnancy and prenatal genetic diagnosis was postponed until 32 weeks in order to minimize the risk of miscarriage. It revealed a balanced female karyotype carrying the paternal translocation. Birth occurred at week 39 and was uncomplicated.

## 3. Conclusion

We showed that DGC could effectively reduce the proportion of unbalanced spermatozoa in chromosomal translocation and pericentric inversion carriers. We, therefore, started a multicentric program in which couples with a male translocation are offered intrauterine inseminations, even in the absence of infertility. We report here the first birth obtained through this program. Further research should aim at finding a way to accurately select chromosomally normal gametes for fertilization in those patients.
